# Childhood fever: a qualitative study on parents’ expectations and experiences during general practice out-of-hours care consultations

**DOI:** 10.1186/s12875-015-0348-0

**Published:** 2015-10-07

**Authors:** Eefje G P M de Bont, Nicole Loonen, Dagmar A S Hendrix, Julie M M Lepot, Geert-Jan Dinant, Jochen W L Cals

**Affiliations:** Department of Family Medicine, CAPHRI School for Public Health and Primary Care, Maastricht University, PO Box 616, 6200 MD Maastricht, The Netherlands

**Keywords:** Child, Infection, Antibiotics, Family practice, Primary health care, Qualitative research

## Abstract

**Background:**

Fever in children is common and mostly caused by benign self-limiting infections. Yet consultation rates in primary care are high, especially during GP out-of-hours care. Therefore, we aimed to explore experiences of parents when having visited GP out-of-hours services with their febrile child.

**Methods:**

We performed a qualitative study using 20 semi-structured interviews among parents from different backgrounds presenting to GP out-of-hours care with a febrile child <12 years. Questions were directed at parental motivations, expectations and experiences when visiting the GP out-of-hours centre with a febrile child. Interviews were audio-recorded, transcribed and analysed using constant comparison technique.

**Results:**

We identified four main categories emerging from the data; (1) cautiously seeking care, (2) discrepancy between rationality and emotion, (3) expecting reassurance from a professional and (4) a need for consistent, reliable information. Not one symptom, but a combination of fever with other symptoms, made parents anxious and drove care seeking. Although parents carefully considered when to seek care, they experienced increased anxiety with increases in their child’s temperature. Because parents work during the day and fever typically rises during the early evening, the decision to seek care was often made during out-of-hours care. When parents consulted a GP they did not have any set expectations other than seeking reassurance, however a proper physical examination diminished their anxiety. Parents did not demand antibiotics, but trusted on the expertise of the GP to assess necessity. Parents requested consistent, reliable information on fever and self-management strategies.

**Conclusions:**

Parents were inexperienced in self-management strategies and had a subsequent desire for reassurance; this played a pivotal role in out-of-hours help seeking for childhood fever. These factors provide clues to optimise information exchange between GPs and parents, by providing written, tailored, consistent information on self-management strategies for current and future fever episodes. GPs’ had incorrect assumptions that parents expected antibiotic treatment.

**Electronic supplementary material:**

The online version of this article (doi:10.1186/s12875-015-0348-0) contains supplementary material, which is available to authorized users.

## Background

Fever is the most common reason for a child to be taken to the general practitioner (GP) and, in the absence of the safety net of their own GP, out of hours care [1, 2]. Still, it is largely unknown what parents expect when consulting out of hours care and only limited evidence about what drives these consultations exists. Previous studies were quantitatively structured using mainly closed questions or performed in a different setting like an emergency department or the general public [3–9]. In an emergency department setting it is likely that children are more seriously ill than in a primary care setting, thereby influencing parental worries and decisions. Additionally, most consultations for childhood fever take place in primary care. Despite this, one in three GP out-of-hours consultations for children are fever related and more than 92 % of these children are managed by GPs without referral to secondary care [10]. The low referral rate highlights the general self-limiting nature of childhood fever in general practice. Despite of this, one in three children with a fever receives an antibiotic when visiting the GP out-of-hours centre [11, 12].

Insight into expectations and experiences of parents who have consulted with out-of-hours GPs with their feverish child could provide insights for future interventions targeted at increasing parental self-management, decreasing the number of (re-)consultations and potential overuse of antibiotics for febrile children during GP out-of- hours care where most febrile children are evaluated. Therefore, it is important to understand why parents consult a GP out-of-hours, what they generally experience and expect, and how they use and would desire information to be given before, during and after a consultation for childhood fever.

This qualitative descriptive study aimed to provide an in-depth overview of these factors, by exploring parental motivations, expectations and experiences with GP out-of-hours consultations for childhood fever.

## Methods

We performed a qualitative study based on naturalistic inquiry using semi-structured interviews to study parents’ expectations and experiences towards consultations with their febrile children at a GP out-of-hours centre [13]. We applied the consolidated criteria for reporting qualitative research (COREQ, see Additional file 1) and adhered to RATS guidelines for reporting qualitative research [14].

### Setting

GP out-of-hours services in the Netherlands are organized in large-scale cooperatives [15]. These cooperatives cover the primary care by rotating shifts of GPs during evening, nights and all weekends. All out-of-hours services in the Netherlands have a triage centre in which trained nurses conduct telephone triage under supervision of a GP and divide all contacts into either telephone advice, GP consultation or home visits by GPs. More than 95 % of GPs provide out-of-hours care through this system.

The study was carried out at a large GP cooperative in Heerlen, the province of Limburg, the Netherlands. The Nightcare GP out-of-hours service in Heerlen, located in a multi-ethnic, moderate to low socio-economic area, is a Dutch GP out-of hours service providing care to approximately 270.000 inhabitants [16].

### Participants

All parents presenting to the GP out-of-hours centre with a febrile child under the age of 12 years in November 2013, were eligible for inclusion and were asked prior to consultations to participate in a semi-structured interview. Parents were approached at the desk of the GP out-of-hours centre by a member of staff and asked to voluntarily sign up. We used purposive sampling based on gender, age, parity, education level and cultural background. There were no exclusion criteria. Low educational level was defined as vocational school or lower, intermediate as higher national diploma or Bachelor’s degree and high as a Master’s degree or higher.

### Data collection

Data were collected between November 2013 and January 2014. An interview guide was prepared using sensitizing concepts [17]. Questions were derived from existing literature and a priori expert discussion. The questions were directed at parental motivations, expectations and experiences when visiting the GP out-of-hours centre with a febrile child. A pilot study consisting of two, 1 h lasting focus groups, facilitated by an experienced and independent moderator, were performed to check for face validity. Based on this pilot study minor changes were made to the interview guide. The purpose of these focus groups was to test the interview guide, therefore they were not used during the analysis.

Based on the adapted interview guide, three trained researchers (DH, NL and JL) conducted face-to-face semi-structured interviews. The interviews, which lasted around 30–45 minutes, were conducted in the participants’ homes or at the GP out-of-hours centre depending on the preference of the parent, within 2 weeks after the consultation. Since their children were sick at the moment they were approached, we believed it would be unethical and undesirable to perform the interview immediately after the consultation. Data saturation was achieved after 14 interviews but to ensure maximum variation 20 semi-structured interviews were performed. The interviews were audio recorded and transcribed verbatim by DH, NL and JL.

### Analysis

Data were analysed using constant comparison technique, coding and analysing took place simultaneously [18]. Inductive analysis was used, by using open and finally axial coding schemes using NVivo software version 9.0 [19, 20]. Inconsistencies about coding were discussed and resolved by consensus.

### Trustworthiness

Data triangulation was enhanced by using parents of different ages, education levels, gender and parity and socio-economic areas and combining interview transcripts with research diaries. Hereby we were able to recruit fathers as well. Previous studies suggested fathers frequently play an important role in decision making about consultation of a doctor [21]. In addition, all researchers used a research diary to take notes on their observations and ideas about the interviews and coding scheme. This was used during interpretation and coding of the interviews and peer debriefings with the whole research team. Furthermore, a member check of the written transcript was performed among all participating parents. We provided detailed information about the methodology and background information of the parents, to help others decide whether the results are transferable to their context.

### Ethical considerations

All parents received written information and provided written informed consent to participate in this study. Data were used anonymously. The study was approved by the Medical Ethics committee of the Maastricht University Medical Centre in Maastricht (ref. number NL 13-4-060.4).

## Results

Of 63 parents who visited the GP out-of-hours service with a febrile child were approached in person, 51 parents consented to receive more information about participating. From these 51, 6 parents participated in the pilot study and 20 parents participated in a semi-structured interview. Of the parents who participated in the semi-structured interviews, there were 7 fathers and average age was 32.2 years (range 22–44 years). None of the parents wanted to alter the transcript after the member check. In Table 1 the main characteristics of the participating parents are described.

**Table 1:** Characteristics of the participating parents (*n* = 20)

Characteristics of parents	Number of parents
Male sex, *n* (%)	7 (35)
Age (years)	
20–29	8
30–39	7
40–49	5
Civil state	
Single parent	1
Living together/Unmarried	6
Married	13
Number of children	
1	9
2	7
3	4
Native country	
The Netherlands	16
Germany	1
Turkey	1
Morocco	1
South-Africa	1
Education	
Low	17
Intermediate	2
High	1
Working parents	15

**Fig. 1:**
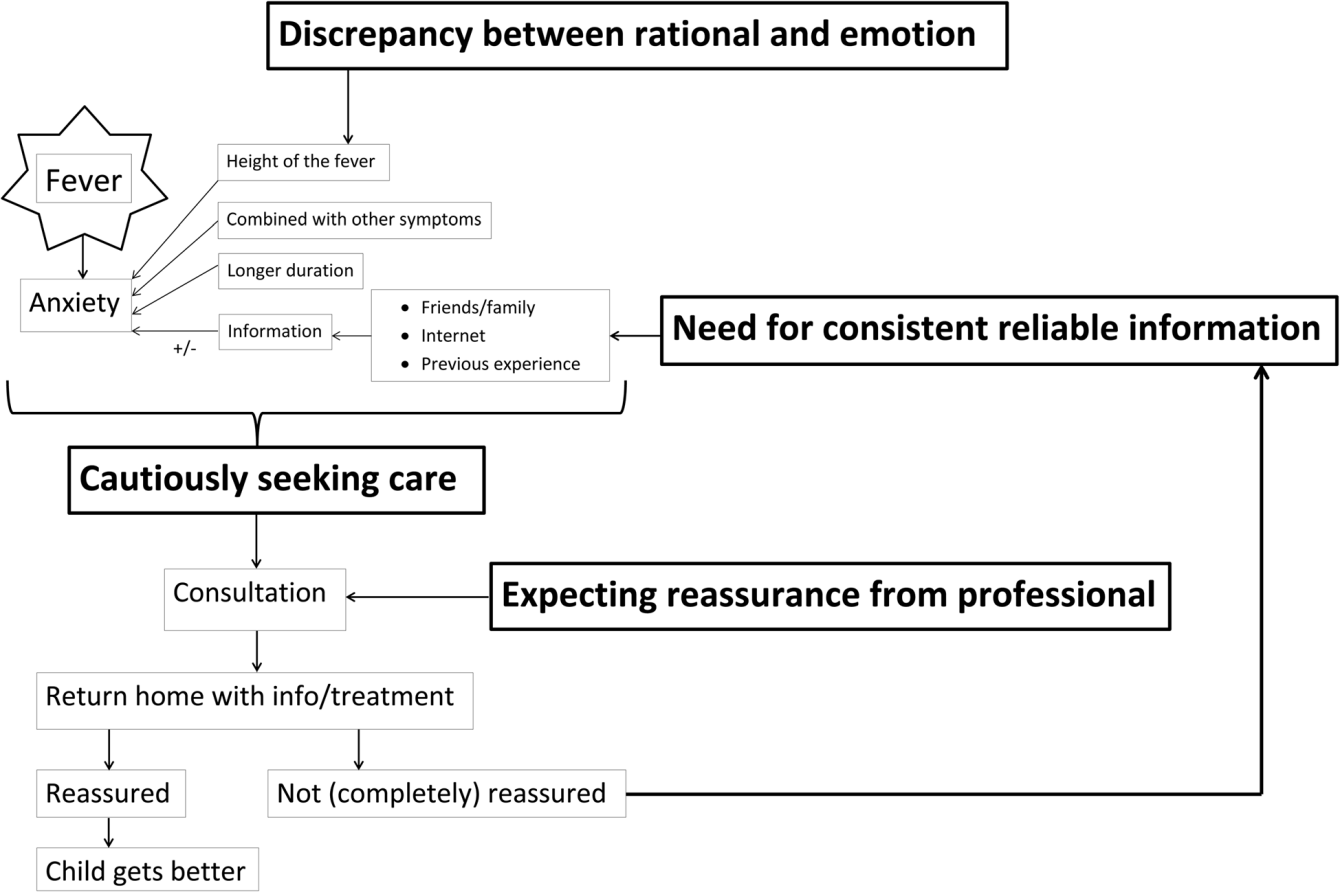
Categories and their relationship. *Main categories are put in Bold*

We identified four main categories emerging from the data; (1) cautiously seeking care, (2) discrepancy between rationality and emotion, (3) expecting reassurance from a professional and (4) a need for consistent, reliable information. These main categories will be discussed in further detail. We did not observe any distinct differences between parents of different gender, age or education level. Figure 1 shows a graphical overview of the categories that were found.

### Cautiously seeking care

Parents expressed a range of experiences and emotions caring for their sick child before even considering contacting the GP out-of-hours service. Contacting the GP out-of-hours service is not prompted by the fever itself, but mostly by a combination of symptoms. The additional symptoms were felt to be the main drivers of their worries and subsequently would then lead to help-seeking behaviour. Specific symptoms named by parents were listlessness, weepiness, sleepiness, lower intake of fluids and their child behaving differently than normal.

*“If he’s still playing I’m not worried. But last time I called the GP because he refused to eat, he wouldn’t drink, he wouldn’t play, he just laid there listlessly on the couch, and that is not my son.” (I5)*

Parents reported ongoing fever, fever not responding to antipyretics and the duration of the fever as important factors influencing their decision to contact the GP out-of-hours service.

*“If it’s only a short fever peak, I’m not worried. But when it [the fever] perseveres for hours, or days, I think that is disturbing.” (I1)*

Parents said they usually carefully wait and see before actually seeking care, especially during out-of-hours care. When they decided to seek care many described that nothing could persuade them from wanting to see a doctor at that point and that was their main reason for contacting the GP out-of-hours centre and not their own GP.

*“Nobody could have stopped me. And nobody could have said to me: no, you do not need to come over right now, just visit your own GP tomorrow.” (I5)*

Factors influencing this decision were on one side logistical like the fact that their own GP didn’t have time that same day, they picked up their child from day-care when their own GP practice was already closed or they wanted to avoid putting the child in the car in the middle of the night.

*“Then we picked him up [their child at day care] and my girlfriend called the GP and they didn’t have time for an appointment anymore and told us to come over the next day. But I didn’t want to wait that long.” (I19)*

On the other side there were also other factors which were related to the fact that they experienced that fever most often rises in the early evening [22]. Parents expressed they were afraid to go to sleep when their child has a fever because they can’t monitor their behaviour then.

*“I noticed the fever was 39.4 °C at the beginning of the evening, which means it will only increase and I’m afraid to go to sleep then.” (I6)*

### Discrepancy between rationality and emotion

Although parents carefully considered when to seek care, they experienced that their anxiety increases with the temperature of their child.

*“…if the temperature is 39 °C I think okay, but if it rises towards 40 °C … I panic.” (I13)*

This may be explained by the fact that most parents believed the height of the temperature directly correlates with the illness severity of their child*.* They explained that in their eyes, a higher temperature correlated with a more serious illness and therefore a higher risk of complications.

*“If it is higher than 40 °C, then you are talking about another, yes another severity.” (I4)*

However, there were also parents who acknowledged that their emotions would often take over from rational reasoning at these instances.

*“Rationally I think it does not matter 38, 39 or 39.5 °C, but emotionally I feel the higher the fever, the sooner something [complications] might happen.” (I7)*

This discrepancy between rationality and emotion was also something that was reported in relationship to a longer duration of illness.

*“Initially I usually don’t panic… but if the symptoms last longer I’m not as sensible and I tend to get on the emotional side of the story.” (I1)*

Underlying, this was possibly related to the fact that parents were willing to await spontaneous improvement to a certain extent. However, if symptoms lasted longer they sought reassurance that they were correctly caring for their child.

*“The GP reassured me at that moment [during a consultation when symptoms lasted for more than three days]. He reassured us that we were on the right track and we just had the keep going caring for our child the way we were doing. That my child would recover by himself.” (I4)*

The only influencing background characteristic of the participating parents we found was the fact that experienced parents (i.e. with older children) were less anxious, perhaps because they had experienced uncomplicated infections and fever with their other children and hence perceived these symptoms as a normal part of childhood.

*“I also hear this from other parents. That it developed like that. They also say, I was a mess backthen. And now, with my second child, it’s okay. I’m not that afraid anymore.” (I10)*

### Expecting reassurance from a professional

The next category that came forward when elaborating on reasons to contact the GP out-of-hours service was the desire to be reassured. The first reassurance as mentioned in the previous category was the reassurance that they were caring for their child in a correct way and there was nothing else that they could do. Some parents just wanted general reassurance from a professional. Parents would formulate this in in different ways.

*“…when the GP says, it’s okay, then it’s okay.” (I10)*

Others specifically wanted to know the cause of the fever and reassurance that their child did not suffer from a serious illness, and was therefore not at risk of complications.

*“…It is looking for confirmation, having the idea that it’s nothing serious… And having that confirmed by a doctor.” (I8)*

When asked what the most reassuring aspect of a GP’s consultation was, the most often mentioned contributing factor was a physical examination by a GP.

*“The GP successfully reassured us. He performed a good physical examination and took us seriously. I believe that’s important.” (I7)*

The position of a GP as an expert in advice on how to care for their child’s health was also described by parents when they were asked about their expectations of medication prescriptions. Parents did not expect medication, but relied on the expertise of the GP to determine what was needed. None of the parents expected antibiotics.

*“…We prefer no medication. But when it is necessary, so when the GP advises medication, we would have given this.” (I7)*

During GP out-of-hours care in the Netherlands, patients are most likely to be seen by another GP than their own GP during daytime care. Yet, seeing a different GP does not bother them, as long as their child is seen by a doctor who takes them seriously and who can treat their child correctly.

*“A doctor is a doctor, he [my child] just needs help.” (I13)*

Parents noticed that they are used to frequently seeing another doctor during daytime care because they attend a GP group practice. Moreover, some parents experienced a consultation with a different doctor as positive considering it as a second opinion.

### A need for consistent, reliable information

Parents consulted other parents or relatives, as well as the internet as an information source before contacting the GP out-of-hours service. Conversely, this did not necessarily lead to reassurance. Some parents even pointed out searching for information on the internet led to increased anxiety.

*“Sometimes I search for information on the internet, but as I have just said, I try to avoid it most of the time… Because I just read too much and then I get anxious.” (I1)*

Most parents did not receive written information from the GP during the consultation. Some parents suggested that information about alarm symptoms and self-management strategies would be helpful when they return home since they did not think about all the questions they had at that exact moment during a consultation.

*“…Written information would be helpful. Because, most often during a consultation you do not think about all the questions you want to ask. And once you are at home you think to yourself hmmm (sighs) I should have asked that…” (I1)*

Additionally, parents described they could use this information for future illnesses before contacting the GP out-of-hours service.

*“For example he diagnosed an ear infection and I believe this is common in small children. If there was a booklet describing this, what an ear infection is and with a picture of the localisation…I would keep that. And I would take a look at it if he gets another ear infection. To recall what’s going on in case I don’t completely remember.” (I13)*

However, parents experienced that it would be important that this information came from one, comprehensible and reliable information source without inconsistencies.

“*There is a need for one good information source which is clear and consistent, not in doctor language because normal human beings don’t understand that.” (I6)*

## Discussion

### Main findings

Parents generally cautiously wait and see before contacting GP out-of-hours care when their child has a fever. Not one specific symptom, but a combination of fever with other symptoms, makes them anxious and drives care seeking. Because parents work during the day and fever typically rises during the early evening, the decision to seek care was often made during out-of-hours care. When contacting a GP out-of-hours service, parents did not expect antibiotics but sought reassurance from a professional, which parents felt could be achieved by a thorough physical examination. Finally, they believed that there is a lack of reliable consistent information on (self-management strategies for) childhood fever before, during and after a consultation.

### Comparison with existing literature

The degree of fever alone has previously shown to have a low predictive value for the severity of an illness in children [1]. Interestingly parents mentioned a higher temperature would indicate a more severe illness, sometimes even mentioning a specific limit. This belief is in accordance with previous research, indicating that parents still believe high fever is harmful [2, 23, 24]. There are studies that show that by increasing parental knowledge this misconception can be reduced [25]. However, some parents were able to describe that their fears were not based on their rational knowledge but mostly on their emotions. This means there were also parents who acknowledged they rationally knew that a higher temperature did not indicate a more severe illness, but emotionally their anxiety increased when the body temperature increased. Some secondary care studies describe that despite increasing knowledge, anxiety remains [26]. However, to our understanding this is the first study in which parents actually described this discrepancy between rational and emotions themselves. Education and information about fever might therefore only reduce anxiety to a certain extent. This is something to take into account when developing interventions to reduce anxiety among parents of febrile children.

The need for reassurance from an expert was expressed by all parents and is in accordance with other literature [3, 27, 28]. This reassurance can, at least partly, be obtained by a physical examination. Previous research already showed a physical examination is valued as an important component of a GP consultation and parents feel reassured when they know what’s going on with their child [3, 9, 27, 28]. Parents rely on a GP for their expertise, as reported in a Scandinavian study which was aimed at studying when parents with an ill child consult a physician [9, 27].

In accordance with existing literature, we found that parents’ expectations of a GP’s consultation were not specific and parents generally do not expect antibiotics [3]. However, in agreement with a recent study our data suggests that parents consult because of a perceived threat to their child’s health, which then in turn prompts clinicians to prescribe antibiotics [29, 30]. In contrast, only a small percentage of children presenting with a fever actually requires treatment based on the incidence of serious bacterial infections [31]. However, recent Dutch studies show that one in three to four children with fever who visit the GP out-of-hours service receive antibiotics [10, 11], suggesting that antibiotic prescribing is still higher than warranted. As in adults with acute cough, one explanation could be that GPs assume patients or in this case parents expect antibiotics [32]. This study underlines the fact that parents indeed do not expect antibiotics.

Parents actively search for information before contacting a GP [27]. As suggested previously, we found that this information did not always reassure parents, but even raised anxiety in some cases [2]. A relatively new finding of this study in comparison to previous research is the usage of the internet as a main source of information for parents. One of the challenges in the usage of internet as an information source is the fact that parents expressed that there is a lack of reliable consistent information on the internet [33].

Another important aspect of information provided to parents of febrile children that is suggested in previous studies is the fact that reliable, consistent information can potentially provide parents with better knowledge [34] and with a safety net [7]. By providing parents information on what to do and when to consult when their child has fever in accordance with the NICE 2013 traffic light system [1], their self-management can be increased without leading to complications for their children [7]. As suggested earlier, this may very well be even more effective if the same information that is provided at the point-of-care during a consultation, is also provided to parents in the general public before their children get sick [2].

### Strengths and limitations

This is the first qualitative describing study enrolled in a GP out-of-hours setting that gives in-depth insight into the motivations, expectations and experiences of parents when they visit a GP out-of-hours with their febrile child. This setting is important because most consultations are handled by GPs without referral to secondary care and many consultations take place during out-of-hours care [10].

Despite efforts to make parents feel comfortable and safe by letting them choose the location of the interview, parents may have given socially acceptable answers, thereby holding back valuable information. Because interviews were not executed immediately after the consultation there was some risk of recall bias. We did however perform the interviews within 2 weeks and feel this was the most ethical and pragmatic approach since it is undesirable to execute an interview with parents’ their sick child being present. Although we attempted to describe the motivation, expectations and experiences of parents, there may be potential underlying and influencing factors, which were not discovered during this study.

The different perspectives, member check, peer debriefings and investigator and data triangulation helped to increase trustworthiness. However, all researchers had a medical background with an interest in general practice and infections which might have influenced their views and interpretation of the data. As only parents who visited the out-of-hours service were included, we are missing data from parents who stayed at home with their febrile child. It is possible these parents have different expectations and experiences considering fever. Additionally, since health care systems and illness experience are culturally different, we do not know to what extent these results are generalizable to other countries. Nevertheless, we believe that these results are at least to some extent generalizable to Western countries with similar health care systems. In addition, we provided information about the methodology and background information of parents to help others decide whether the results of this study are transferable to their context.

### Implications for practice

Lacking self-management strategies seem to influence parental consultations which do then in turn potentially thrive antibiotic prescriptions [29, 30]. It is previously shown that an information exchange tool is effective in reducing the number of antibiotic prescriptions and intention to re-consult in children with upper respiratory tract infections and that such a tool can increase parental and clinician confidence in managing these illnesses [21, 35]. We believe that this strategy could also be used in children presenting with a fever. Therefore, future research should focus on improving information on childhood fever provided in the consulting room in a consistent, tailored, written way. However, this might be challenging during out-of-hours care where there is no pre-existing relationship between GPs and parents and where time is limited [30]. Therefore, we believe that future studies should also focus on providing consistent parental education to parents in the general public, thereby improving parental confidence and self-management when their child has a fever.

## Conclusions

Parents of febrile children are still anxious and search for reassurance from a GP as a professional when fever is accompanied by other symptoms. They sought reassurance that they were correctly caring for their child and were additionally reassured when a thorough physical examination was conducted. This study demonstrates, in accordance with previous research, that parents of a febrile child do not expect antibiotics and are in search of consistent, reliable information about fever and specific symptoms. Enhancing parental knowledge may provide parents with a safety net [7], thereby influencing self-management and the parental need for consultations. In addition, by making this information available in the consulting room it may facilitate communication about caring for a febrile child and address misconceptions GP’s still hold about parents and patients expecting antibiotics.

## Additional file

Additional file 1:
**Consolidated criteria for reporting qualitative studies (COREQ): 32-item checklist.** (DOCX 22 kb)

## Abbreviations

GP: General practitioner
COREQ: Consolidated criteria for reporting qualitative research
