# Supplementary material for: Childhood fever: a qualitative study on parents’ expectations and experiences during general practice out-of-hours care consultations
Source: BMC Fam Pract. 2015 Oct 7;16:131. doi: 10.1186/s12875-015-0348-0 (PMC4597376; doi:10.1186/s12875-015-0348-0)
Supplement: Additional file 1: — Consolidated criteria for reporting qualitative studies (COREQ): 32-item checklist. (DOCX 22 kb) [file 12875_2015_348_MOESM1_ESM.docx]

**Consolidated criteria for reporting qualitative studies (COREQ): 32-item checklist**

| **No** | **Item** | **Guide questions/description** |
| --- | --- | --- |
| **Domain 1: Research team and reflexivity** |  |  |
| *Personal Characteristics* |  |  |
| 1. | Interviewer/facilitator | Which author/s conducted the interview or focus group?  *Eefje G.P.M. de Bont, MD*  *Nicole Loonen, MD*  *Dagmar A.S. Hendrix, MD*  *Julie M.M. Lepot, MD* |
| 2. | Credentials | What were the researcher's credentials?  *See question 1* |
| 3. | Occupation | What was their occupation at the time of the study?  *Medical doctor/student and researchers* |
| 4. | Gender | Was the researcher male or female?  *4 female, 2 male* |
| 5. | Experience and training | What experience or training did the researcher have?  *All researchers were trained in interview techniques and performing qualitative research* |
| *Relationship with participants* |  |  |
| 6. | Relationship established | Was a relationship established prior to study commencement?  *No* |
| 7. | Participant knowledge of the interviewer | What did the participants know about the researcher? e*.g. personal goals, reasons for doing the research*  *The participants received written information about the purpose of the study prior to the interview.* |
| 8. | Interviewer characteristics | What characteristics were reported about the interviewer/facilitator?  *In the discussion, we mention the fact that all researchers had a medical background with an interest in general practice and infections which might have influenced their views and interpretation of the data.* |
| **Domain 2: study design** |  |  |
| Theoretical framework |  |  |
| 9. | Methodological orientation and Theory | What methodological orientation was stated to underpin the study?  *We performed a qualitative study based on naturalistic inquiry using semi-structured interviews, constant comparative technique and sensitizing concepts.* |
| Participant selection |  |  |
| 10. | Sampling | How were participants selected?  *We used purposive sampling based on gender, age, parity, education level and cultural background. There were no exclusion criteria.* |
| 11. | Method of approach | How were participants approached?  *All parents presenting to the GP out-of-hours centre with a febrile child under the age of 12 years in November 2013, were eligible for inclusion and were asked face-to-face prior to consultations to participate in a semi-structured interview.* |
| 12. | Sample size | How many participants were in the study?  *Of 63 parents who visited the GP out-of-hours service with a febrile child were approached in person, 51 parents consented to receive more information about participating. From these 51, 6 parents participated in the pilot study and 20 parents participated in a semi-structured interview.* |
| 13. | Non-participation | How many people refused to participate or dropped out? Reasons?  *12 parents refused to participate because of a lack of time for an interview.* |
| Setting |  |  |
| 14. | Setting of data collection | Where was the data collected?  *Interviews were performed in participants’ their home.* |
| 15. | Presence of non-participants | Was anyone else present besides the participants and researchers?  *During some interviews the child or other spouse was present but not participating in the interview.* |
| 16. | Description of sample | What are the important characteristics of the sample?  *The characteristics are displayed in table 1.* |
| Data collection |  |  |
| 17. | Interview guide | Were questions, prompts, guides provided by the authors? Was it pilot tested?  *An interview guide was prepared using sensitizing concepts. Questions were derived from existing literature and a priori expert discussion. The questions were directed at parental motivations, expectations and experiences when visiting the GP out-of-hours centre with a febrile child. A pilot study consisting of two, one hour lasting focus groups, facilitated by an experienced and independent moderator, were performed to check for face validity.* |
| 18. | Repeat interviews | Were repeat interviews carried out? If yes, how many?  *No* |
| 19. | Audio/visual recording | Did the research use audio or visual recording to collect the data?  *Audio* |
| 20. | Field notes | Were field notes made during and/or after the interview or focus group?  *Yes, research diaries* |
| 21. | Duration | What was the duration of the interviews or focus group?  *30-45 minutes* |
| 22. | Data saturation | Was data saturation discussed?  *Yes* |
| 23. | Transcripts returned | Were transcripts returned to participants for comment and/or correction?  *Yes a member check was performed* |
| **Domain 3: analysis and findings**z |  |  |
| Data analysis |  |  |
| 24. | Number of data coders | How many data coders coded the data?  *Four* |
| 25. | Description of the coding tree | Did authors provide a description of the coding tree?  *Yes, see figure 1* |
| 26. | Derivation of themes | Were themes identified in advance or derived from the data?  *Derived from the data* |
| 27. | Software | What software, if applicable, was used to manage the data?  *NVivo software version 9.0* |
| 28. | Participant checking | Did participants provide feedback on the findings?  *All participants responded that they agreed on the findings and had no further feedback.* |
| Reporting |  |  |
| 29. | Quotations presented | Were participant quotations presented to illustrate the themes / findings? Was each quotation identified?  *Yes* |
| 30. | Data and findings consistent | Was there consistency between the data presented and the findings?  *Yes* |
| 31. | Clarity of major themes | Were major themes clearly presented in the findings?  *Yes* |
| 32. | Clarity of minor themes | Is there a description of diverse cases or discussion of minor themes?  *Yes* |
